# *“When my mother called me to say that the time of cutting had arrived, I just escaped to Belgium with my daughter”*: identifying turning points in the change of attitudes towards the practice of female genital mutilation among migrant women in Belgium

**DOI:** 10.1186/s12905-020-00976-w

**Published:** 2020-05-19

**Authors:** Afi A. Agboli, Fabienne Richard, Isabelle Aujoulat

**Affiliations:** 1 Faculty of Public Health, Université Catholique de Louvain, 30 Clos Chapelle aux Champs, 1200 Brussels, Belgium; 2 GAMS Belgium (Groupe pour l’Abolition des Mutilations Féminines), Brussels, Belgium

**Keywords:** Female genital mutilation, Turning points, Migrant women, Patriarchal system, Emotions

## Abstract

**Background:**

Female Genital Mutilation (FGM) is a public health concern with negative consequences on women’s health. It is a harmful practice which is recognized in international discourses on public health as a form of gender-based violence. Women are not only victims of this, but also perpetrators. The practice of FGM remains a social norm which is difficult to change because it is deeply rooted in tradition and is embedded in the patriarchal system. However, some women have managed to change their attitudes towards it and have spoken out against it. This study identifies and describes *turning points* that have been defined as significant and critical events in the lives of the women, and that have engendered changes in their attitudes towards the practice of FGM.

**Methods:**

We have conducted an inductive qualitative study based on the life story approach, where we interviewed 15 women who have undergone FGM. During the interviews, we discussed and identified the turning points that gave the research participants the courage to change their position regarding FGM. The analysis drew on lifeline constructions and thematic analysis.

**Results:**

Six common *turning points* relating to a change in attitude towards FGM were identified: *turning points* related to (i) encounters with health professionals, (ii) education, (iii) social interactions with other cultures and their own culture, (iv) experiences of motherhood, (v) repeated pain during sexual or reproductive activity, and (vi) witnessing the effects of some harmful consequences of FGM on loved ones.

**Conclusions:**

The *turning points* identified challenged the understanding of what it means to be a ‘member’ of the community in a patriarchal system; a ‘normal woman’ according to the community; and what it means to be a ‘good mother’. Moreover, the turning points manifested in conjunction with issues centered on emotional responses and coming to terms with conflicts of loyalty, which we see as possible triggers behind the shift experienced by the women in our sample.

## Background

Female Genital Mutilation is defined as all procedures whereby the external female genitals are removed for non-therapeutic reasons [1]. The practice is performed mainly in sub-Saharan Africa as well as in the Middle East, Indonesia and Malaysia [2]. It is estimated that two hundred million women and girls have been subjected to the practice worldwide [2]. International migration brought the practice to other parts of the world and it has become a global public health concern to host countries [3]. FGM is a harmful practice due to: (i) its consequences on women’s health; (ii) the violation of women’s bodily integrity, as a healthy organ is cut without a medical reason. Complications related to FGM vary from both immediate to long-term concerns and sometimes require interventions from health professionals [4]. Several studies have looked at the negative consequences of FGM on the lives and health of women who have undergone it [1, 5, 6]. The immediate consequences include pain, severe hemorrhage, urine retention and urinary tract infections. The long-term impacts include depression, Post-Traumatic Stress Disorder (PTSD), and difficulties in relation to painful sexual intercourse [1].

The practice of FGM suggests gender-based violence, a violation of women’s health rights and of human rights generally [1, 7]. As FGM is mainly performed on young children, it violates the rights of children and undermines those of girls to health, security and physical integrity [1, 8].

The practice of FGM involves a whole community, making it a social norm that everyone is expected to comply with [1]. In some FGM-practicing communities, FGM is a celebratory rite of passage which reinforces cultural and ethnic identity and a sense of belonging in the community [1, 9]. Individuals and families believe their communities expect them to do it in order to ensure an honorable and worthy womanhood for their daughters [1]. Cultural cues reinforce the social significance of FGM, and the practice is maintained for the strong meanings attached to it: virginity, beauty and purity, rite of passage to womanhood, and marriageability [1, 10]. The practice as a norm is also reinforced through several other norms that are embedded in the patriarchal system. These norms include the submissiveness of girls throughout their childhood, roles of childbearing and rearing as well as the sexual satisfaction of men [11]. Grandmothers and mothers have the responsibility to uphold the tradition and to perpetuate the practice on their daughters. Girls are taught to be brave, endure pain, and not to express their emotions. The practice is often perpetrated upon women and girls by other women [10, 12]. The related norms embedded in the patriarchal system together with the associated meanings of performing FGM make the practice resistant to change.

### The change of attitudes towards the practice of FGM

Despite the normative system that makes the change difficult, some women still succeed in changing their attitudes towards the practice. Most research about the change of attitudes towards FGM has been conducted at a community level, and only to a much lesser extent at individual level. In order to understand how communities may succeed in changing their attitudes, these studies used different perspectives, such as the human rights approach and anti-FGM campaigns [13]; the legislative perspective [1, 14–16], the social convention perspective [17, 18] and the behavioral approach to change [13, 17, 19].

Approaches based on the human rights perspective and anti-FGM campaigns have been mainly adopted by Non-Governmental Organizations (NGOs). They used human rights messages and communicated negative health consequences of FGM to emphasize the harmful repercussions of the practice, and to convince communities to change their attitudes and stop cutting their daughters. However, such campaigns failed to make a distinction between particular health complications associated with different types of FGM, and communities did not view all types of FGM as responsible for adverse consequences [13, 20, 21]. As a result, their efforts to eliminate all forms of FGM were undermined.

The legislative approach was used to criminalize the practice of FGM either as a specific criminal act or as an act of general bodily harm. Studies found that environmental factors in contexts where the practice is against the law influence the change in attitudes among migrants [18, 22]. O’Neill et al. [23] assert that the length of time spent in host countries is associated with the change of attitudes towards the traditional practice.

The social convention approach was used to suggest that the eradication of these practices may be achieved through renouncing it publicly [18]. The public renouncement of communities was meant to make families believe it to be acceptable and not detrimental to their status not to cut their daughters [13, 24]. There is some evidence that this approach may be successful in the short-term. For example, in Senegal, a whole community collectively declared their renunciation of FGM [24]. In the long-term however, the change is hardly sustained, as some of the women excisors, although they had renounced the practice publicly, ‘had gone back to their scissors’ some years later in order not to lose their economic status [24, 25].

With regards to the behavioral change approach, intervention programs have applied the stages of change or the Transtheoretical Model (TTM) [26] to FGM, with the aim of achieving a change of attitudes at the community level. This remained challenging, as the decision to cut a girl is beyond the parents’ power, and often involves several individuals, including father, mother, grandmother, aunts, and potential in-laws [25]. According to some authors who have applied the Transtheoretical Model of change to FGM at a community level, their approach failed to address important individual dimensions in the dynamics of community change [27].

And thus, in all these approaches, the impact of interventions aimed at changing attitudes towards FGM was mostly studied at the community level. How change occurs at the individual level still remains an under-investigated issue, which our study seeks to address by researching critical events in the lives of women, that led to a personal change of attitudes towards the practice of FGM.

Researching critical life events or *turning points* to understand changes in personal attitudes has proven particularly relevant when studying sensitive issues, such as overcoming intimate partner violence [28–30], quitting drug use [31], or criminal offending [32].

Through the identification of common *turning points*, this paper looks at what makes individual migrant women in Belgium, who were once socialized in the FGM cultural context where the practice is valued and normal, change their attitudes towards the practice and speak out against it.

## Methods

Qualitative methodology informed by the life story narrative approach was used to investigate, identify and describe critical life events experienced by the women in their change of attitude towards the practice of FGM. Life stories research uses the concept of *turning points* to describe changes in the life trajectory of individuals [32]. Wheaton and Gotlib [33] claim that *turning points* can only be found in the context of life trajectories, and they define them as specific events perceived to change the direction of one’s life [34]. These can only be identified in hindsight after the event has passed, and thus are subjective and retrospective reconstructions of life story narratives [35]. In the life course perspective, the events are revealed as something that helps people to change status from disadvantaged to successful, from criminal to non-criminal, from abused and battered to breaking out of the relationship and becoming free [36]. Embedded in one’s life story, *turning points* are shifts that force individuals to recognize that they are no longer who they used to be [37].

This study focuses on *turning points* as significant events which create an awareness that challenges the existing internalized norms in relation to the practice of FGM.

### Sample (participants)

Fifteen women who self-reported that they had once undergone FGM and now stood against it were included in the sample. They were considered eligible to participate if they self-reported to have undergone FGM, were 18 years or over, had been living in Belgium for at least 1 year, were from an FGM-practicing community from East or West Africa, spoke either French or English and self-reported as being against the practice of FGM. Ten women were recruited through gatekeepers from a non-profit organization, GAMS-Belgium *(Groupe pour l’Abolition des Mutilations Sexuelles féminines),* which strongly opposes FGM. This initial convenience sample was followed by a snowball procedure that led to the inclusion of a further five women. All the women participants we recruited came from five different countries in sub-Saharan Africa and provided written informed consent in order to participate. The informed consent process included an overview of the objectives of the study, and an appointment was set by mutual agreement for an interview at a place that suited each woman. We also mentioned their ongoing rights as participants and reassured them that they were free to stop without having to explain why. Given the sensitivity of the topic, we had anticipated the possibility that they could give a pseudonym when signing the consent form, but they all gave their real names. The recruitment process took place between December 2016 and April 2017.

The age of the women participants varied from 23 to 53 years old, with a median age of 39. The age when FGM was performed varied from 5 to 14, with a median age of 7. There was a range of women from across East (33%) and West (67%) Africa. They had been living in Belgium for a median duration of 6 years. The other characteristics are presented in Table 1.

**Table 1:** Summary of participants characteristics at the time of the interview (*n* = 15)

Variables	N (47)	Median/Range
**Age of the woman at time of interview**		39 years [23–53]
**Age when FGM was performed**		7 years [5–14]
**Region of origin**:		
East Africa	5 (33%)	
West Africa	10 (66%)	
**Level of education:**		
Primary school	1 (0.6%)	
Secondary school	7 (46%)	
University	7 (46%)	
**Occupation**		
Unemployed	5 (33.33%)	
Voluntary work	4 (26.66%)	
Student	6 (40%)	
**Length of stay in Belgium at time of interview**		6 years [1–15]
**Method of entry to Belgium**:		
Asylum	13 (86.66%)	
Family reunion	2 (13.33%)	
**Marital status at the time of the interview**:		
Married to an African man (same community)	6 (66.66%)	
Married to an African man (other community)	1 (11.11%)	
Married to a Belgian	2 (22.22%)	
**Divorced once but now cohabiting with a Belgian at time of interview**	6 (40%)	
**Total number of children at time of interview**	25	
**Number of children per woman**		2 [1–4]
**Number of children born in Belgium**	15 (60%)	
**Woman giving birth to at least 1 girl**	11 (44%)	
**Woman with 1 child left in the country of origin**	1 (0.4%)	

#### The iterative process of data collection and data analysis

In-depth interviews were conducted by the first author, and each woman was interviewed twice in the Biographical Narrative Interview Method (BNIM) developed by Wengraf [38] in order to produce narratives relating to life events. BNIM draws on several theoretical perspectives to take a case-based approach to narrative analysis [38]. Within the BNIM approach to data collection, the interviewee is seen in two phases and sometimes three, with the first interview being unstructured and the consecutive interviews building on the previously collected data.

Phase One starts with a single statement which is known as ‘a single question aimed at inducing narrative (SQUIN)’ [38]. Interviewees are encouraged to talk freely about their life stories in the way they decide and without interruption, allowing memories to surface and connections between thoughts to develop. In Phase Two, researchers review their field notes for all topics mentioned by the participants to develop further narratives around them. The second phase generates rich data around incidents prompted by the researcher from which the respondent could choose. Phase Three within BNIM is not always present in studies but does allow an opportunity for the researcher to follow up on more specific points [38] and to be more structured with questioning, should this be appropriate.

### First interview

Our first interviews were conducted either at the GAMS offices (*n* = 8) or in the women’s own homes (*n* = 7), according to the women’s preferences. The interviews lasted from 30 min up to an hour and a half, with an average of 45 min. An explanation of the objectives of the study was provided to the women before the beginning of the interview so that they would know that FGM would be discussed. Then, at the start of the interviews, the women were all asked this one, broad question as suggested by Wengraf [38] and Bertaux [39]: *Could you tell me about your life experiences, and in doing so, include any story in your life that you think important?* The women were encouraged to talk freely about their lives and to tell as much or as little of their story as they wanted. The women’s stories were recorded, and their consent was sought beforehand. Only one woman refused to be recorded, so notes were taken.

This first interview enabled the analysis to start by constructing lifelines for each woman. If a woman specified or emphasized an event during the interviews, that event was considered significant. A ‘lifeline’ is a visual depiction of a life story which displays events in chronological order and also shows the importance of events [40]. We drew along an *x axis* with events entered in chronological order, in such a way that the main events were visually represented along with the link to any environmental context. Figure 1 (Additional file 1) shows a ‘lifeline’ from a fictive vignette of a typical reconstructed story from different participants after the first and second interviews.

Hypotheses of *turning points* were thus inferred from life stories in relation to existing norms embedded in the patriarchal system and associated with FGM, such as: *keeping virginity, beauty and purity*, *ensuring the rite of passage to womanhood and marriageability*, *ethnic identity, being subordinate, and the acceptance of pain* and *suffering that women must endure without complaint.* These represented our predefined categories.

### Second interview

The second interview was conducted in the women’s own homes and lasted from 50 min to 1 h and 40 min with an average of 1 h and 15 min. Within this length of time, the women were able to: (i) confirm the hypotheses of *turning points* raised after the first interview, (ii) narrate more events and complete the lifelines, and (iii) identify further relevant *turning points*, if any. The second interview was guided by semi-structured questions that were unique to each woman according to their initial narratives. Additional file 2 shows the interview guide with general questions. This enabled us to complete and validate the lifelines with every woman, with a visual representation of the significant events (*turning points*) that led to a change of attitudes towards the practice of FGM. After the confirmation of individual’s *turning points*, we pursued with a comparative analysis of all the transcripts, case sheets and lifelines in order to identify common categories of *turning poin*ts across the range of life narratives. In doing so, we noticed that some of the *turning points* overlapped, so we grouped them again in accordance with those similarities. All emerging themes were discussed throughout between the first and last author, involving the second author whenever possible without breaking confidentiality. We moved back and forth to rearrange the groupings, until consensus was reached on six categories of *turning points*. These are as follows: *Turning points related to encounters with health professionals; education; social interactions with other cultures and their own culture; motherhood and the urge to protect daughters; repeated pain during sexual or reproductive activity; and witnessing the effects of some harmful consequences of FGM on loved ones.*

### Ethical considerations

During the recruitment and before the beginning of each interview, the women were verbally given information, including the objectives of the study. We stated to them that their participation in the study was voluntary and that because we were aware of the sensitivity of the topic, they may withdraw from the study at any time. They were also told that the information obtained in this research might be published in a scientific journal, but that their identity would be kept strictly confidential. They were assured that all data would be kept locked in the student’s office and destroyed after the PhD thesis would be completed.

They agreed, and all signed the written consent form in their own names even though an option was given to them to sign with a pseudonym. The study received approval from the Ethics Committee *(Comité d’Ethique Hospitalo-facultaire)* of Saint Luc University Hospital- Brussels with reference number: 2013/21NOV/522; dated: July 10, 2017.

## Results

In reporting our results, we shall first illustrate how the women in our study had internalized the practice of FGM as a social norm, before reporting on the turning points that led to a change of attitude and the decision to take action in their lives.

### Attitudes towards FGM as a mandated social norm before the *turning points*

The women in our sample confirmed that FGM is indeed a powerfully enforced norm, which they used to be forbidden to speak about. It was considered taboo, and they could not discuss it with their siblings. They reported that they were forbidden by their grandmothers to look down at or touch their private parts. However, their mothers were proud to show off to new members of the community after the procedure. Some women reported that they had asked to undergo FGM, to avoid being mocked by peers and to be allowed to serve men tea and food. They also believed they would be considered clean, hygienic, more beautiful and likely to keep their virginity for marriage. This was believed to preserve the family honor and morality of girls and women.

*“ … At home we did not talk about it; it was taboo. We were forbidden to tell others what had happened ... Nobody spoke about how it happened ... a girl must be excised otherwise she will not be a virgin, so she will always run after men. She cannot control herself; she will run after all the men she will meet and so we must go through that to preserve our virginity and not be unfaithful after marriage ... So, virginity has a lot of weight in that sense.” Interv_6*

*“I have undergone female circumcision and I asked for it because I was fed up with being excluded from playing with my friends … it was normal, because your mother, grandmother, your aunt, and neighbors have all undergone it and everything is normal” Interv_8.*

The other related norms embedded in the patriarchal system were for the elders to be obeyed and the grandmothers to be the guardians of the tradition, forced marriage, the way women ought to behave in the community, and that women must endure pain and suffering without complaining. Most women explained that after FGM, forced marriage would follow. Gender roles were carefully reinforced, either by their mothers or their grandmothers: for instance, how a woman ought to behave in the community and be submissive to her husband, and how they should endure pain and be brave.

*“We were told all sorts of things, such as how to behave later with our husbands, how to respect them, the good manners that a good wife should always have and always listen to them. We were told that a girl has to go through that, and we should pass it on to the next generation. They have gone through it, so we have to go through it too. That’s how it is, it’s a custom to be respected...” Interv_2.*

One woman recounted how she was given a white sheet by her father as a gift on the day she was cut, despite expecting sweets and toys as she was only 6 years old. When she asked about it, she was told that it was for her wedding. She was later forced to marry an old man whom she met only on the wedding day.

*“My dad chose someone I did not even know, an old man, far older than me and I was forced to marry him … it's very difficult, (silence) because it's something that stays with you ... because you are being raped. I do not call that marriage, it’s a rape … ” Interv_1*

### The main *turning points* that led to changes in women’s lives

#### *Turning points* related to an encounter with health professionals

These turning points concerned events where the women encountered a health professional: a gynecologist, a psychologist, or a social worker. For example, during gynecological visits, the women reported that they were shocked to be told that they did not have a normal vulva and were shown the intact anatomy of the vulva of their daughters, which was different from their own. This led them to understand the difference between an intact vulva and one that has been mutilated, as well as some negative consequences of the practice of FGM. They also mentioned that this shock led to the awareness that what they had thought was a ‘normal’ vulva (one that was *“pure and beautiful”* after FGM) was not. Other women mentioned that they were surprised, confused, and felt anxiety at the news of what an intact vulva looked like. One participant had been persuaded that all women, including white women, were like her. The picture shown by the doctor brought on an understanding of the organ that had been lost and led participants to question what it means to be a ‘normal woman’. For some women in our sample, this led to taking action for a deinfibulation procedure. Others, at the time of the interview, were considering having a reconstruction of the clitoris.

*“So I went to see a gynecologist at a family planning clinic. She put me on the table and examined me and said you're cut and closed … She put my daughter on the table too and showed me, you see she is not cut, she is intact not like you … So, for the first time I saw the difference between my daughter and myself” Interv_11*

*“ … When you visit a gynecologist, you are surprised when the doctor tells you that you are not ‘normal’. With the expression of his face ... he looks and looks; he closes his eyebrows and says to you like this: you're not normal … and I was confused and anxious ... And you realize, after explanation with photos, the difference between the normal and abnormal private part. So, I say, I have never seen the thing between the legs … ” Interv_8*

#### *Turning points* related to education

This type of *turning point* involves events such as lectures on anatomy and sexuality at school and university, where some women, enrolling at medical school and attending anatomy lectures, started changing their views. Schools and universities have been eye-openers. The knowledge gained resulted in the feelings of shock and anger experienced by most women, and this made them change their attitudes towards the practice. The anatomy lectures contributed to the knowledge of the consequences of FGM and what the normal anatomy of a woman ought to be.

*“But during my studies, I realized some things and it was a shock … The first time I saw the genital organ of a woman, I said ah ... so I lost this part of me in the excision ... But hey, it's a bit what like I looked as well. But it must be said that this operation is very traumatic. We only perpetuate the tradition of our ancestors. All you gain is pain and sorrow.” Interv_13*

#### *Turning points* related to social interactions

These *turning points* relating to social interactions are two-fold. One is in relation to **interactions with other cultures** and involved events where the women heard the noise of urine at refugee centers, got married, or had a relationship with a European man when they came to Belgium. Migrating from their country of origin to Belgium contributed to raised awareness of differences between cultures, and a sense of not being defined exclusively by FGM. The shock provoked by the noise of urine coupled with the women noticing that “*women are urinating like men”* made them question something that they had previously thought was normal. They no longer viewed women as having to endure pain and suffering when men from other communities made them aware of the possibility that their sexual lives could be experienced without pain or complication during intercourse.

*“When you come here, you discover that not all women are like you. Because you see women go to the bathroom, and their pee makes a noise ... (laughs). So, I asked myself ‘What have they got there?’ And I asked my doctor once, ‘You're not circumcised?’ She says ‘No’ … then I understood why their pee makes noise.” Interv_8*

The second interaction was **within the women’s own culture** when they were told of the reasons why FGM is performed, for them not to be promiscuous before marriage, and they saw the opposite happening around them in the community. This made them realize the lies and the deceit.

*“On the one hand I saw that it was false, that we were told lies because I saw Fulani women who prostituted themselves, and I asked myself some questions ... these circumcised girls prostitute themselves – how does it happen? ... I also saw some circumcised girls who became pregnant before marriage and brought shame upon their families.” Itnerv_2*

#### *Turning points* related to motherhood and the urge to protect their daughters

The women in our sample wanted the best for their children. Those of them who had girls reported that at some point or other they had been put under pressure by mothers, mothers-in-law and grandmothers or aunts, the keepers of the tradition, to put their own daughters through FGM. The pressure from other women in their families made them recall their own experiences and brought back vivid memories of the whole procedure. Some talked about pain in the womb, anger, nightmares and the urgent need to fly away to escape the danger. The prospect of perpetuating the tradition on to the next generation through their own mothers, mothers-in-law and grandmothers triggered a change in views about the practice for several women, creating a sense of apprehension as well as a duty to protect their daughters, which in turn changed their views of what it means to be a good mother. According to their previous beliefs, a good mother would put her own daughter through FGM. After becoming mothers, themselves, they did not want to put their daughters through what they had experienced. They were caught in a dilemma of loving both their mothers and their daughters, and therefore, disappointing their mothers by not wanting to destroy their daughters’ lives through FGM.

*“ … My husband could not say ‘no’ to his mother, and it had become very serious, something had to be done to protect my daughter from the influence of my mother-in-law ... I tried to tell him we shouldn’t listen to his mom for everything and he answered me, “Aren’t you yourself excised? So why not your daughter? You see?” And I did not want that for my daughter … ” Interv_4*

*“I was destroyed by my mother and my grandmother. I can say that since they have done something horrible to me ... I love them but when my in-laws wanted to excise my daughter, as was usual. But I opposed.” Interv_15*

Another woman reported that she lied to her mother, telling her that she had performed FGM on her daughter at the hospital. However, the grandmother found out 3 years later and informed the mother. The mother pressured the daughter over the phone. As a result, the daughter fled abroad.

*“My mother called me to say with a lot of pressure that the time for her daughter has arrived to be made pure and clean, … I took a radical action without thinking and escaped abroad” Interv_11*

#### *Turning points* related to repeated pain during sexual and reproductive activity

The sexual and reproductive aspects identified as *turning points* were mainly associated with repeated pain, childbirth and sexual activity: pain felt during the procedure of FGM when the participants were little girls; painful monthly periods as adolescents; pain during their first experience of sexual intercourse after their marriage; and pain during childbirth. The repeated pain during sexual intercourse implied that the women took part in it out of duty towards their husbands, rather than for pleasure. They used to think that experiencing pain during the sexual intercourse was normal until they developed an awareness of what sexual activity could be. They then came to understand the real consequences of FGM.

*“But then what we do not understand is how much it hurts ... it's horrible, and it follows you everywhere ... even in adulthood, in your teenage years when menstruating, when you get married, when you have sex with your husband, if you give birth, if you go through a caesarean … you see? The pain follows you everywhere, and it's horrible.” Interv_8*

*“ … I had convinced myself that I would not be able to have a fulfilling sex life, and I was right because when I got married, it opened the door to another phase of a woman's life of suffering ... It gives no benefit, just suffering and I find that men also suffer, not only women.” Interv_10*

#### *Turning points* related to witnessing the effects of some harmful consequences of FGM on loved ones

These *turning points* are related to events that happened to the women’s loved ones and that gave rise to stressful emotions for them. For instance, the participants in our sample reported events such as the death of a sister after the procedure, witnessing their husbands being battered by their own families for not wanting to comply with the tradition and the death of a sister in childbirth. Such events made the women realize the harm caused by FGM.

*“ … Because after our excision, we stayed with an old woman for 20 to 30 days, but my sister only made it for six days. She had a high fever, and she bled a lot and the old woman kept changing cloths and made her drink various concoctions until she died the following day … ” Interv_12*

## Discussion

### Understanding the significance of *turning points* (TPs) in the changing of attitudes towards the practice of FGM

This paper identified and described *turning points* defined as significant and critical events which created an awareness that challenged the norms embedded in the patriarchal system and associated with the practice of FGM. The *turning points* in the lives of the women who participated in our study occurred as a result of events where the women either encountered health professionals or attended educational settings and through that education became aware of the normal anatomy of the female genitals. Moreover, experiences of motherhood were reported when pressure from their mothers-in-law, their mothers and grandmothers made them question what it meant to be a good mother to their daughters. How the experience of fearing for one’s children is associated with making decisions to change correlates with other studies on *turning points* but is related to other forms of violence against women, for example, intimate partner violence [28, 41].

Another type of event related to *turning points* was found to be linked to social interactions within one’s own culture or with other cultures. The events related to reproductive and sexual activity included pain during menstruation, childbirth, and repeated pain during sexual intercourse. As other authors have reported about *turning points* associated with issues other than FGM, the *turning points* found in our study created either ‘sudden’ awareness from a single event or ‘gradual’ awareness from repeated events [28, 42, 43].

### Challenging what it means to be a member within the community and a normal woman

The different *turning points* that led to a change of attitudes towards FGM in our study frequently challenged the norms of what it means to be a ‘normal’ woman in the eyes of the community, and what it means to be ‘a member of the community’ in a patriarchal system*.* The community dictates what a ‘normal woman’ is supposed to be and do. For example, a ‘normal woman’ is supposed to be cut, to behave in a certain way in the community, to be a virgin before marriage, to endure pain and suffering, and not to show emotions. Also, a ‘normal woman’ does not experience any sexual desire or pleasure. If a girl is cut, she is a full member of the community.

The norms related to FGM, which were embedded in the patriarchal system, and which were challenged by the women, made them more conscious of the gender roles their communities had projected on them. Challenging these interconnected norms is somehow challenging the “invisible cage” imposed by the gender roles the patriarchal system has established [10]. They came from communities where both girls and boys were taught these gendered relationships to power throughout their lives. This explains why the women used to see the practice of FGM, as well as other related norms, as ‘normal’. However, their consciousness of these gender roles evolved through events in the women’s lives that caused them to begin to question the legitimacy of what they used to consider ‘normal’. The realization of what the women considered to be simple everyday life was challenged and changed by what now constitutes for them a ‘normal woman’.

Their consciousness of gender roles gave them a platform from which to acquire new knowledge through turning points, which was added to the knowledge gained during childhood. For Lawrence and Valsiner [44], new information integrated into an individual’s previous understanding makes the individual either focus on or reject the new information. The women in this study focused on the new information and came to learn, for example, the *normal anatomy of female genitals* (new information); they then processed it and internalized it into new knowledge *(normal anatomy)*. The new knowledge, in this case, helps to challenge what has been internalized in childhood. Lien and Schultz [45] researched the internalized knowledge with the change of attitudes about FGM among migrant women in Norway. They found that some women activists had undergone FGM and seen it as normal yet had later changed their attitudes towards the practice. What they had internalized as normal was processed into a new knowledge through exposure to negative consequences of FGM and preceded an attitudinal change [45].

While recalling critical events associated with *turning points* in their lives, the women in our study expressed emotions. Thus, it appears that new knowledge happened through the recognition of experiencing certain emotions such as anger, shock, and astonishment when they acknowledged the normal anatomy of female genitals. They experienced the same in various educational settings. Moreover, astonishment, surprise, and loss of trust were seen in their social interactions. At the same time, empathy, flashbacks of their own experiences and sadness were identified in the *turning points* related to experiences of motherhood as well as when they witnessed the effects of some harmful consequences of FGM on their loved ones. Yet, the right to the recognition and expression of their own emotions is something that had until then been denied to these women, as they were raised and taught in their communities as young girls that it is normal for women to endure pain and suffering without complaining.

### Challenging what it means to be a good mother within the community

The *turning points* in our study also challenged the norm of what it means to be a ‘good mother’, as good mothers according to the norms in the community are expected to ensure their own daughters meet all requirements of the patriarchal system, including the practice of FGM. In this case, being a ‘good mother’ meant that they did not want to put their daughters through FGM. As they wanted to do good by their daughters by not putting them through FGM, the desire to protect their daughters made them experience ambivalent and uncomfortable feelings towards their own mothers. Indeed, they wanted to hate their mothers for putting them through FGM, but at the same time, they understood that their mothers had wanted somehow the best for them.

We therefore hypothesize that *turning points* that generated some emotions may be associated with conflicts of loyalty which the women needed to come to terms with if new values and norms were to be internalized. Being a ‘good mother’ is therefore connected to the existing core value of caring for children*.* Mackie [46] put it well in saying that the most important, fundamental, and personal value of parents worldwide is to take good care of their children and protect them from harm. When, in our case, mothers were put under pressure (for example, phone calls to put a daughter through FGM, or the decision of a mother-in-law to excise the woman’s daughter), they did not necessarily change what Mackie identifies as their basic values [46]. Instead, the basic value, e.g. ‘being a good mother’ was reinforced but took on a new meaning and therefore, a new outcome.

### Strengths and limits

There are several limits to our study: our sample of 15 women is relatively small, and some sub-groups of women may be under-represented or over-represented. For instance, none of the women in our sample were single mothers, and half of them were cohabiting with or were married to a Belgian man at the time of the interviews. Due to our snowball procedure, there might have been a selection bias in our sample, and we cannot disregard the possibility that other *turning points* might have emerged from further interviews with other women. Moreover, although the main researcher (who is also the interviewer) originates from an African FGM-practicing country herself, difficulties and challenges were still encountered in the recruitment of the women and even during some of the interactions, as we felt that the women had probably censured themselves at times. For these reasons, we cannot be sure that we have reached saturation in our results.

Another source of bias may be linked to our initial recruitment procedure through GAMS-Belgium, and the fact that the first interviews for some women were conducted on GAMS-Belgium premises. However, the second interviews for all the women were conducted in their own homes, thus minimizing the risk of desirability bias. As far as the process of analysis is concerned, all emerging themes were discussed throughout between the first and last author, involving the second author whenever possible without breaking confidentiality. This collaborative process of analysis is one of our study’s strengths. However, the main strength of our research lies in the co-construction of the findings with the women themselves, as these women were involved in the meaning-making process and were invited to confirm the *turning points* that had made them change their attitudes. Due to our rigorous analytical approach and the fact that we allowed the women to co-construct our findings with us through repeated and participative interviews, we believe that our results are trustworthy and transferable enough to be shared with the scientific community.

## Conclusions

This study confirmed that FGM is indeed a social norm through the women’s own words. It also identified *turning points* which enabled the researcher to find norms embedded in the patriarchal system, which went on to be challenged. Coming to terms with the taboo of having emotions and feelings on the one hand, and on the other, the conflict of loyalty that inevitably arises when one questions the legitimacy of the rules and norms of one’s own community, are major challenges that may be seen as common mechanisms for succeeding in changing the attitudes of women who originated from FGM-practicing countries and communities towards FGM. These hypotheses merit further investigation as they may pave the way for further applied research into better understanding the mechanisms by which the women change their attitudes towards the practice of FGM in the context of migration. This might in turn help the women stop perpetuating the practice of FGM and become agents of change within their own communities.

## Supplementary information

**Additional file 1.** A construction of a ‘lifeline’ from a fictive vignette of a typical reconstructed story from different participants after the 1st and 2nd interviews.

**Additional file 2.** Interview guide.

## Data Availability

The datasets generated and/or analyzed during the current study are not publicly available due to confidentiality reasons. The women interviewed shared their personal life stories, and details about what they have been subjected to. We cannot disclose that to the public. However, a de-identified dataset will be made available upon reasonable request of the corresponding author.

## Abbreviations

FGM: Female genital mutilation
TP: Turning points
BNIM: Biographical Narrative Interviewing Method
GAMS-Belgium: *Groupe pour l’Abolition des Mutilations Sexuelles*
NGO: Non-Governmental Organization

## References

[1] Eliminating female genital mutilation: An Interagency Statement: OHCHR, UNAIDS, UNPD, UNECA, UNFPA, UNHCHR, UNHCR, UNICEF, UNIFEM, WHO. 2008. www.who.int/reproductivehealth/publications/fgm. Accessed 15 Jan 2015.

[2] Female Genital Mutilation/Cutting: A global concern. [Internet]. UNICEF. 2016. http://data.unicef.org/topic/child-protection/female-genital-mutilation-and-cutting/. Accessed 10 Apr 2017.

[3] Leye E, Sabbe A. Responding to female genital mutilation in Europe: striking the right balance between prosecution and prevention. Gent: International Centre for Health and Reproductive Health; 2009.

[4] Kimani S, Karibu CW, Muteshi J, Guyo J (2020). Exploring barriers to seeking health care among Kenyan Somali women with female genital mutilation: a qualitative study. BMC Int Health Hum Rights.

[5] Vloeberghs E, Van der Kwaak A, Knipscheer J, Van den Muijsenbergh M (2012). Coping and chronic psychosocial consequences of female genital mutilation in the Netherlands. Ethn Health.

[6] Whitehorn J, Ayonride O, Maingay S (2002). Female genital mutilations: cultural and psychological implications. Sex Relat Ther.

[7] Cook RJ (2008). Ethical concern in female genital cutting. Afr J Reprod Health.

[8] Ahmed HM, Shabu SA, Shabila NP (2019). A qualitative assessment of women’s perspectives and experience of female genital mutilation in Iraqi Kurdistan region. BMC Womens Health.

[9] Farage MA, Miller KW, Tseghai GE, Azuka CE, Sobel JD, Ledger WJ (2015). Female genital cutting: confronting cultural challenges and health complications across the lifespan. Women Health.

[10] Monagan SL (2010). Patriarchy: perpetuating the practice of female genital mutilation. J Altern Perspect Soc Sci.

[11] Alavi R (2003). Female genital mutilation: a capability approach. Auslegung..

[12] Heise L, Manji K (2016). Social norms.

[13] Brown K, Beecham D, Barrett H. The applicability of behaviour change in intervention programmes targeted at ending female genital mutilation in the EU: integrating social cognitive and community level approaches. Obstet Gynecol Int. 2013;2013:1–12. 10.1155/2013/324362 PMC3745976 23983698

[14] Shell-Duncan B, Hernlund Y, Wander K, Moreau A (2013). Legislating change? Response to criminalizing female genital cutting in Senegal. Law Soc Rev.

[15] Johnsdotter S (2019). Meaning well while doing harm: compulsory genital examinations in Swedish African girls. Sex Reprod Health Matters.

[16] United Nations Children’s Fund (UNICEF). Female genital mutilations/cutting: a statistical overview and exploration of the dynamics of change. New York: UNICEF; 2013.

[17] Shell-Duncan B, Wander K, Hernlund Y, Moreau A (2011). Dynamics of change in the practice of female genital cutting in Senegambia: testing predictions of social convention theory. Soc Sci Med.

[18] Mackie G, LeJeune J (2009). Social dynamics of abandonment of harmful practices: a new look at the theory.

[19] Leye E, Bauwens S, Bjakander O (2005). Behaviour change towards female genital mutilation: lessons learned from Africa and Europe.

[20] Hernlund Y, Shell-Duncan B, Hernlund Y, Shell-Duncan B (2007). Transcultural positions: negotiating rights and culture. Transcultural bodies: female genital cutting in global context.

[21] Dustin M (2010). Female genital mutilation/cutiing in the UK: challenging the inconsistencies. Eur J Women's Stud.

[22] Boyle EH, Preves SE (2000). National politics as international process: the case of anti-female genital cutting laws. Law Soc Rev.

[23] O'Neill S, Dubourg D, Florquin S, Bos M, Zewolde S, Richard F (2017). Men have a role to play but they don’t play it: a mixed methods study exploring men’s involvement in female genital mutilation in Belgium, the Netherlands and the United Kingdom: summary.

[24] Diop N, Askew A, Abusharaf RM (2006). Strategies for encouraging the abandonment of female genital cutting: experiences from Senegal, Burkina Faso, and Mali. Female circumcision: Multicultural perspectives.

[25] Toubia NF, Sharief EF (2003). Female genital mutilation: have we made progress?. Int J Gynecol Obstet.

[26] Prochaska JO, Diclemente CO (1983). Satges and processes of sel-change of smoking: toward an integrative model of change. J Consult Clin Psychol.

[27] Shell-Duncan B, Hernlund Y, Wander K, Moreau A (2010). Contingency and change in the practice of female genital cutting: dynamics of decision-making in Senegambia.

[28] Murray CE, Crowe A, Flasch P (2015). Turning points: critical incidents prompting survivors to begin the process of terminating abusive relationships. Fam J.

[29] Chang JC, Dado D, Ashton S, Hawker L, Cluss PA (2006). Understanding behavior change for women experiencing intimate partner violence: mapping the ups and downs using the stages of change. Patient Educ Couns.

[30] Walker K, Bowen E, Brown S, Sleath E (2017). Subjective accounts of the turning points that facilitate desistance from intimate partner violence. Int J Offender Ther Comp Criminol.

[31] Teruya C, Hser Y-I (2010). Turning points in the life course: current findings and future directions in drug use research. Curr Drug Abuse Rev.

[32] Carlsson C (2012). Using turning points to understand processes of change offending: notes from a Swedish study on life courses and crime. Br J Criminol.

[33] Wheaton B, Gotlib IH, Gotlib IH, Wheaton B (1997). Trajectories and turning points over the life course: concepts of themes. Stress and adversity over the life course: trajectories and turning points.

[34] Enz KF, Talarico JM. Forks in the road: memories of turning points and transitions. Appl Cogn Psychol. 2015;30(2):188–95.

[35] Hareven TK, Masaoka K (1988). Turning points and transitions: perceptions of the life course. J Fam Hist.

[36] Reimer D (2014). Subjective and objective dimensions of turning points. Soc Work Soc Int Online J.

[37] Clausen JA, Moen P, Elder GH, Luscher K (1995). Gender, contexts and turning points in adult’s lives. Examining lives in context: perspectives on the ecology of human development.

[38] Wengraf T (2008). Short guide to biographical narrative interviewing and analysis by the SQUIN-BNIM method.

[39] Bertaux D (2016). Le récit de vie.

[40] Adriansen HK (2012). Time line interviews: a tool for conducting life history research. Qual Stud.

[41] Chang JC, Dado D, Hawher L, Cluss PA, Buranosky R, Slagel L (2010). Understanding turning points in intimate partner violence: factors and circumstances leading women victims toward change. J Women's Health.

[42] King G, Cathers T, Brown E, Specht JA, Willoughby C, Polgar JM (2003). Turning points and protective processes in the lives of people with chronic disabilities. Qual Health Res.

[43] Patzel B (2001). Women’s use of resources in leaving abusive relationships: a naturalistic inquiry. Issues Ment Health Nurs.

[44] Lawrence JA, Valsiner J (2003). Making personal sense: an account of basic internalisation and externalisation processes. Theory Psychol.

[45] Lien I-L, Shultz J-H (2013). Internalizing knowledge and changing attitudes to female genital cutting/mutilation. Obstet Gynecol Int.

[46] Mackie G (1996). Ending footbinding and infibulation: a convention account. Am Sociol Rev.

