# Supplementary material for: “When my mother called me to say that the time of cutting had arrived, I just escaped to Belgium with my daughter”: identifying turning points in the change of attitudes towards the practice of female genital mutilation among migrant women in Belgium
Source: BMC Womens Health. 2020 May 19;20:107. doi: 10.1186/s12905-020-00976-w (PMC7236468; doi:10.1186/s12905-020-00976-w)
Supplement: Supplementary file 2 — Additional file 2. Interview guide. [file 12905_2020_976_MOESM2_ESM.docx]

**Interview guide**

*Thank you for your time and participation in this interview. It should last about 1 hour. The aim of the interview is to identify in the life stories the significant events that influenced the change of attitudes towards the practice of FGM.*

**First interview**

Could you tell me about your life experiences, and in doing so, include any story in your life that you think important? You can start wherever you want.

**Second interview**

*The purpose of the second interview is for the women to confirm the hypotheses of turning points, to narrate more events, to identify other relevant turning points and to complete the lifelines together with the women. The second interview was guided by semi-structured questions that were unique to each woman according to their initial narratives.*

-Please could you confirm the hypotheses of *turning points* raised after the first interview?

-Could you tell me more of any event you think that has contributed to any awareness towards the change?

Based each woman’s life story

- What led you to question excision?

-Growing up, what do you think the reason was?

-How do you position yourself regarding FGM now?

-How do you feel about it now?
